# Prognosis for Cutaneous Melanoma by Clinical and Pathological Profile: A Population-Based Study

**DOI:** 10.3389/fonc.2021.737399

**Published:** 2021-11-16

**Authors:** Alessandra Buja, Andrea Bardin, Giovanni Damiani, Manuel Zorzi, Chiara De Toni, Riccardo Fusinato, Romina Spina, Antonella Vecchiato, Paolo Del Fiore, Simone Mocellin, Vincenzo Baldo, Massimo Rugge, Carlo Riccardo Rossi

**Affiliations:** ^1^ Department of Cardiologic, Vascular and Thoracic Sciences, and Public Health, University of Padua, Padua, Italy; ^2^ Clinical Dermatology, Istituto di Ricovero e Cura a Carattere Scientifico (IRCCS) Istituto Ortopedico Galeazzi, Milan, Italy; ^3^ Department of Biomedical, Surgical and Dental Sciences, University of Milan, Milan, Italy; ^4^ PhD Program in Pharmacological Sciences, Department of Pharmaceutical and Pharmacological Sciences, University of Padua, Padua, Italy; ^5^ Veneto Tumor Registry - Azienda Zero, Padova, Italy; ^6^ Surgical Oncology Unit, Veneto Institute of Oncology IOV-Istituto di Ricovero e Cura a Carattere Scientifico (IRCCS), Padova, Italy; ^7^ Department of Surgery, Oncology and Gastroenterology (DISCOG), University of Padua, Padova, Italy; ^8^ Department of Medicine DIMED, Surgical Pathology & Cytopathology Unit, University of Padova, Padova, Italy

**Keywords:** melanoma, clinical characteristics, survival, epidemiology, cancer survival

## Abstract

**Introduction:**

Among white people, the incidence of cutaneous malignant melanoma (CMM) has been increasing steadily for several decades. Meanwhile, there has also been a significant improvement in 5-year survival among patients with melanoma. This population-based cohort study investigates the five-year melanoma-specific survival (MSS) for all melanoma cases recorded in 2015 in the Veneto Tumor Registry (North-Est Italian Region), taking both demographic and clinical-pathological variables into consideration.

**Methods:**

The cumulative melanoma-specific survival probabilities were calculated with the Kaplan-Meier method, applying different sociodemographic and clinical-pathological variables. Cox’s proportional hazards model was fitted to the data to assess the association between independent variables and MSS, and also overall survival (OS), calculating the hazard ratios (HR) relative to a reference condition, and adjusting for sex, age, site of tumor, histotype, melanoma ulceration, mitotic count, tumor-infiltrating lymphocytes (TIL), and stage at diagnosis.

**Results:**

Compared with stage I melanoma, the risk of death was increased for stage II (HR 3.31, 95% CI: 0.94-11.76, p=0.064), almost ten times higher for stage III (HR 10.51, 95% CI: 3.16-35.02, p<0.001), and more than a hundred times higher for stage IV (HR 117.17, 95% CI: 25.30-542.62, p<0.001). Among the other variables included in the model, the presence of mitoses and histological subtype emerged as independent risk factors for death.

**Conclusions:**

The multivariable analysis disclosed that older age, tumor site, histotype, mitotic count, and tumor stage were independently associated with a higher risk of death. Data on survival by clinical and morphological characteristics could be useful in modelling, planning, and managing the most appropriate treatment and follow-up for patients with CMM.

## Introduction

In recent decades, the incidence of cutaneous malignant melanoma (CMM) in white people has been increasing steadily (1, 2). Meanwhile, a significant improvement in CMM patients’ 5-year overall survival has also been reported, and related mostly to the increasing prevalence of cancers detected in their earliest, “thinner” stage” (3, 4). Both the rising incidence of CMM (all stages), and changes in the treatment panorama (also including the advent of targeted therapies) prompt the collection of updated information which might re-orient both prevention efforts and diagnostic/therapeutic strategies.

Based on the natural history of CMM, a well-established set of clinicopathological variables has been significantly correlated with the clinical outcome of melanoma patients. Unfortunately, these data are often inconsistently recorded and/or scattered over different digital archives. This situation interferes with efforts to validate prognostic variables in the “real world” of large-scale population-based studies.

As for the stage-specific survival of CMM patients, most information comes from national cancer registries, and the USA American Surveillance, Epidemiology and End Results program (SEER) in particular (5). To the best of our knowledge, few registry-based studies on the stage-specific survival of CMM patients have been conducted in Italy or elsewhere in Europe in the last two decades (6–10).

The present study investigates the five-year melanoma-specific survival (MSS) for all cases of CMM recorded in 2015 in the resident population of a north-eastern Italian region (Veneto). Both demographic and clinical-pathological variables have been considered to measure their impact on patient survival in this cohort of CMM patients.

## Materials and Methods

### Context

The Italian public national health service (NHS) is financed mainly by general taxation, and is largely managed on a regional basis. NHS policies are grounded on fundamental values of universality, free access, freedom of choice, pluralism in provision, and equity.

In the north-eastern Veneto region of Italy, the Regional Authority has endorsed a number of standardized Diagnostic Therapeutic Protocols (DTPs) for the clinical management of cancer patients. All DPTs have been edited by multidisciplinary task forces including dedicated experts belonging to the Regional Oncology Network (ROV).

This retrospective study on the outcome of CMM patients is based on clinico-pathological information recorded by the Veneto Cancer Registry in 2015 (11).

### Study Participants and Data Collection

This retrospective population-based study involves a cohort of 1,279 incident cases of CMM diagnosed in the Veneto region in 2015 (resident population: 4,915,123). For each patient, the following set of clinical-pathological features were considered: a) tumor site (lower limbs, upper limbs, head, hands/feet, trunk); b) CMM histological subtype (lentigo maligna, acral lentiginous, blue nevus, desmoplastic, nodular, superficial spreading, spitzoid); b) growth phase (radial *versus* vertical); c) histologically-proven ulceration (present *versus* absent); d) number of mitoses (categorized as 0-2 or >2) (12); e) tumor-infiltrating lymphocytes, ([TILs] absent *versus* present; f) TNM stage, as established by merging clinical and pathological information available at the time of patient enrolment (13).

Patients were grouped by age in the following brackets: < 40, 40-49, 50-59, 60-69,70-79, 80 years or more.

### Statistical Analysis

The number of person-years in the cohort was calculated by taking the date of entry as the time when a tumor was diagnosed, and the date of exit as 31 December 2020 or the time of death or drop-out from follow-up, whichever came first. Patient deaths were considered in the overall survival (OS) analysis regardless of their cause, while only deaths caused by melanoma were considered in the analysis of MSS. The cumulative MSS rates were calculated with the Kaplan-Meier method using different sociodemographic and histopathologic features. Cox’s proportional hazards model was fitted to the data to assess the association between both MSS and OS and the previously-detailed independent variables (except for growth type as this variable perfectly predicted the outcome). In the multivariate analysis, we grouped the less common histological categories (acral-lentiginous, blue nevus, desmoplastic, spitzoid) as “Other”. A sensitivity analysis was performed, excluding stage IV patients from the multivariate analysis. The assumption of proportionality was accepted for all models. Statistical significance was ascertained using an alpha level of 0.05 and two-sided tests. All data analyses were run using the R statistical package (version 3.6.3; R Studio, Boston, MA).

### Ethics

The data analysis was performed on anonymous aggregated data with no chance of individuals being identifiable. Ethical approval for the study was obtained from the Veneto Oncological Institute’s Ethics Committee (n. 52/2016).

## Results

In 2015, the Veneto Cancer Registry 1,279 incident CMM-patient were registered at. **Table 1** shows patients’ demographics (M/F: 1.13; median age: 58 years) and clinical-pathological profiles. Most of the invasive malignancies were diagnosed in the early stage (stage I: 71.8%). The mean follow-up was 1,670 ± 415 days.

**Table 1:** Baseline characteristics of the study cohort (NOS, not otherwise specified; TILs, tumor infiltrating lymphocytes).

	Number (%)		Number (%)
**All patients**	1,279 (100)	**Mitotic count**	
**Sex**		0-2	798 (62.39)
Male	678 (53.0)	>2	252 (19.70)
Female	601 (47.0)	Not known	229 (17.91)
**Age (years)**		**TILs**	
<40	155 (12.1)	Present	927 (72.5)
40-49	252 (19.7)	Absent	189 (14.8)
50-59	252 (19.7)	Not known	163 (12.7)
60-69	257 (20.1)	**Tumor status (T)**	
70-79	217 (17)	T1	820 (64.1)
80+	146 (11.4)	T2	167 (13.1)
**Tumor site**		T3	126 (9.8)
Lower limbs	260 (20.33)	T4	98 (7.7)
Upper limbs	195 (15.25)	TX	14 (1.1)
Head	133 (10.40)	Not known	54 (4.2)
Hands/feet	56 (4.38)	**Nodal status (N)**	
Trunk	593 (46.36)	N0	1,119 (87.5)
Not known	42 (3.28)	N1	64 (5)
**Histological subtype**		N2	45 (3.5)
Superficial spreading melanoma	926 (72.40)	N3	31 (2.4)
Nodular melanoma	159 (12.43)	Not known	20 (1.6)
Lentigo maligna	28 (2.19)	**Metastasis status (M)**	
Acral-lentiginous melanoma	25 (1.95)	M0	1,225 (95.78)
Desmoplastic melanoma	4 (0.31)	M1	26 (2.03)
Blue nevus	1 (0.08)	Not known	28 (2.19)
Spitzoid melanoma	28 (2.19)	**TNM Stage (enrolment)**	
NOS Malignant melanoma	34 (2.66)	I	918 (71.8)
**Growth phase**		II	161 (12.6)
Horizontal	285 (22.3)	III	117 (9.1)
Vertical	701 (54.8)	IV	26 (2)
Not known	293 (22.9)	Not known	57 (4,5)
**Ulceration**		Sentinel lymph node (*)	
Yes	202 (15.8)	Performed	360 (0.45)
No	1,003 (78.4)	Not performed	86 (80.35)
Not known	74 (5.8)	Not known	2 (19.20)

(*) Only for patients with stage pT1b-pT4b and on stage I-II.

Overall, the 5-year OS was 83.8% (95% CI: 81.8, 85.8) and it was higher for females (86.6%; 95% CI: 84.0, 89.4) than for males (81.2%; 95% CI: 78.4, 84.2). Five-year MSS was 92.5% (95% CI: 91.0, 94.0), with no significant survival advantage for females (93.6%; CI: 91.7, 95.6) over males (91.5%; CI: 89.4, 93.7).

**Figure 1:**
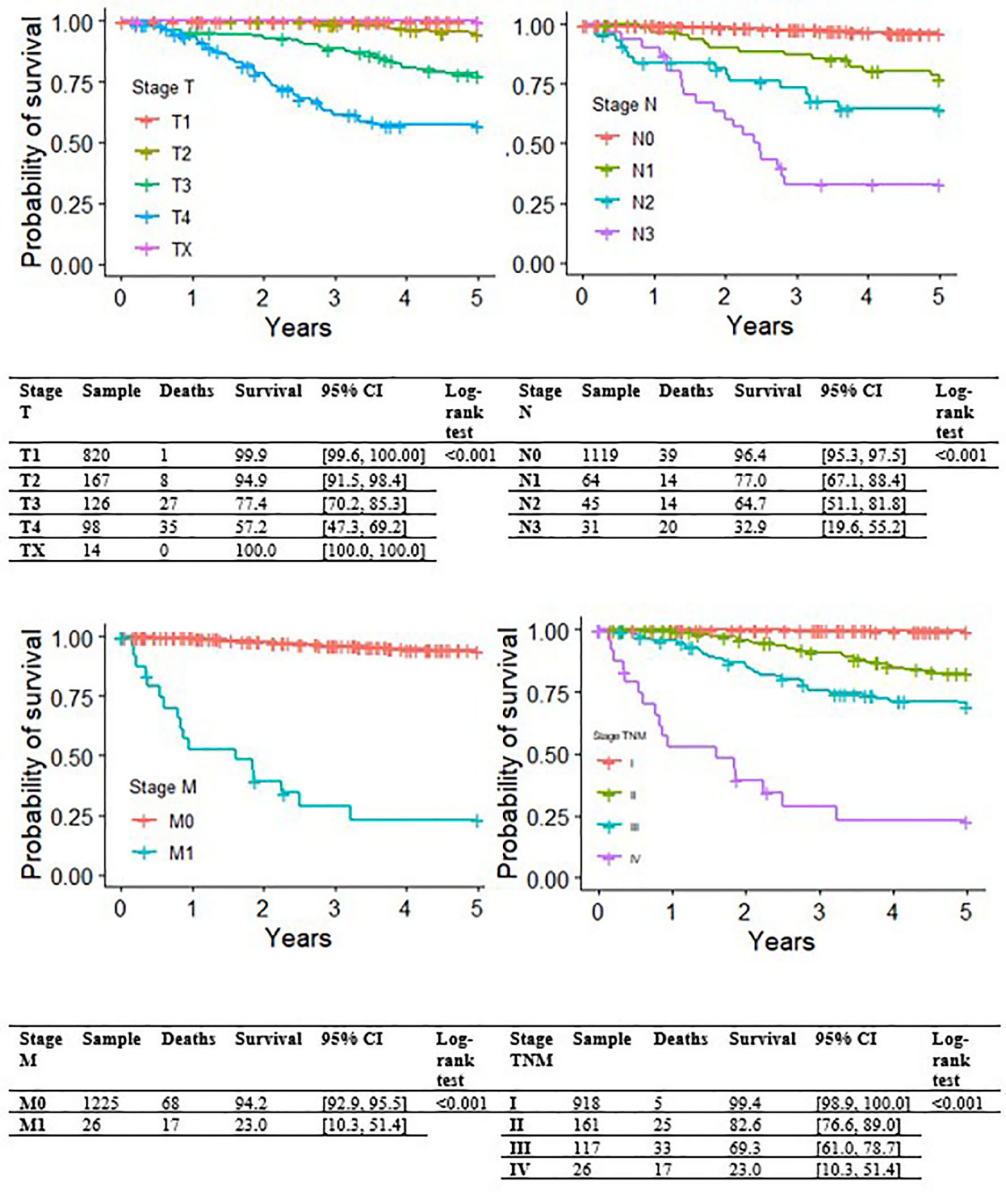
Kaplan-Meier curves for melanoma-specific survival by stage (T, N or M, and TNM overall).

**Figure 1** shows Kaplan-Meier MSS curves by TNM clinical-pathological staging at initial diagnosis, which had a strong impact on survival; T, N and M values are also reported. The 5-year MSS was 99.4% (95% CI: 98.9-100.0) for stage I, 82.6% (95% CI: 76.6-89.0) for stage II, 69.3% (95% CI: 61.0-78.7) for stage III, and only 23.0% (95% CI: 10.3-51.4) for stage IV.

**Figure 2:**
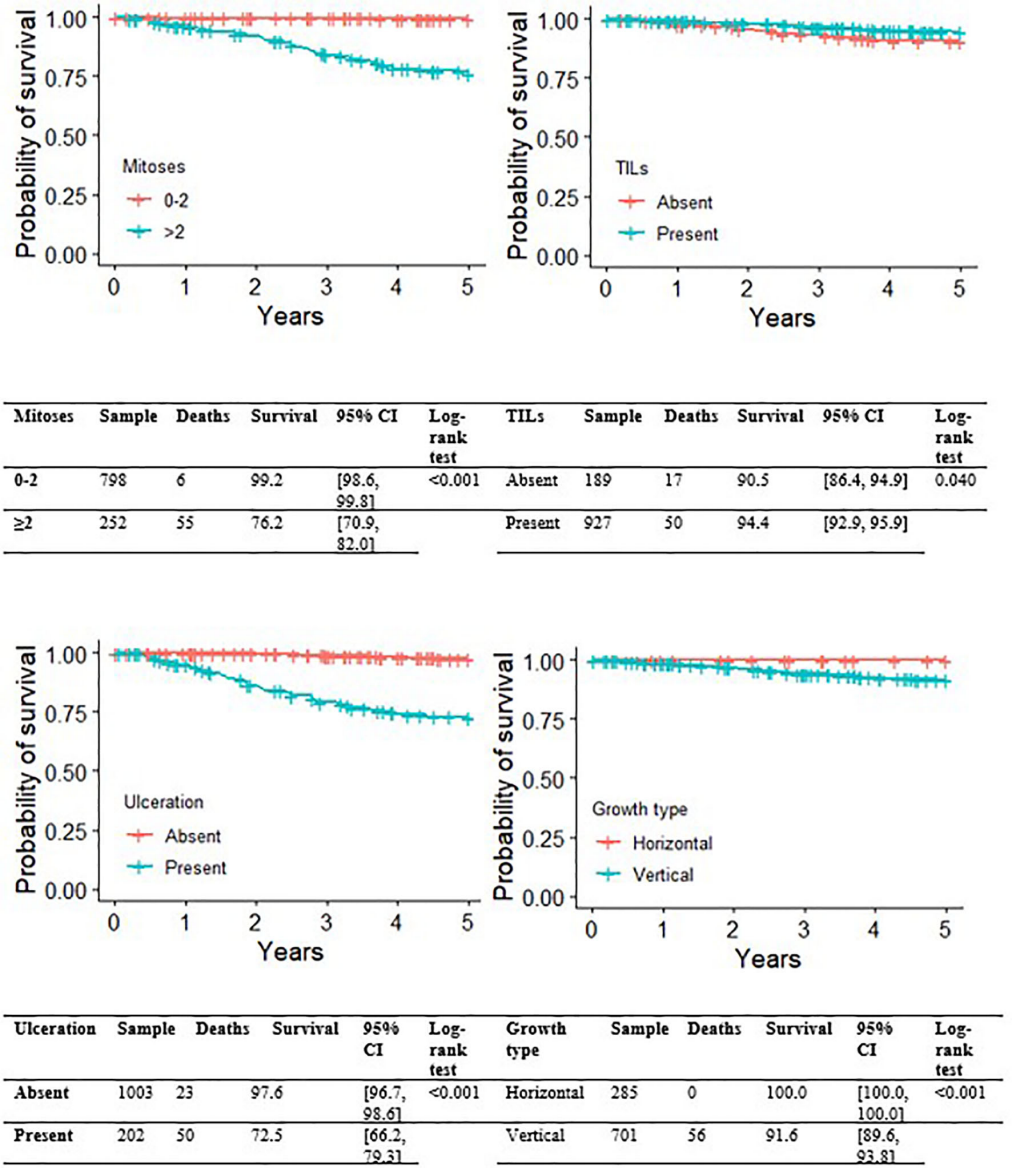
Kaplan-Meier curves for melanoma-specific survival by presence of ulceration, growth phase, presence of TIL.

**Figure 3:**
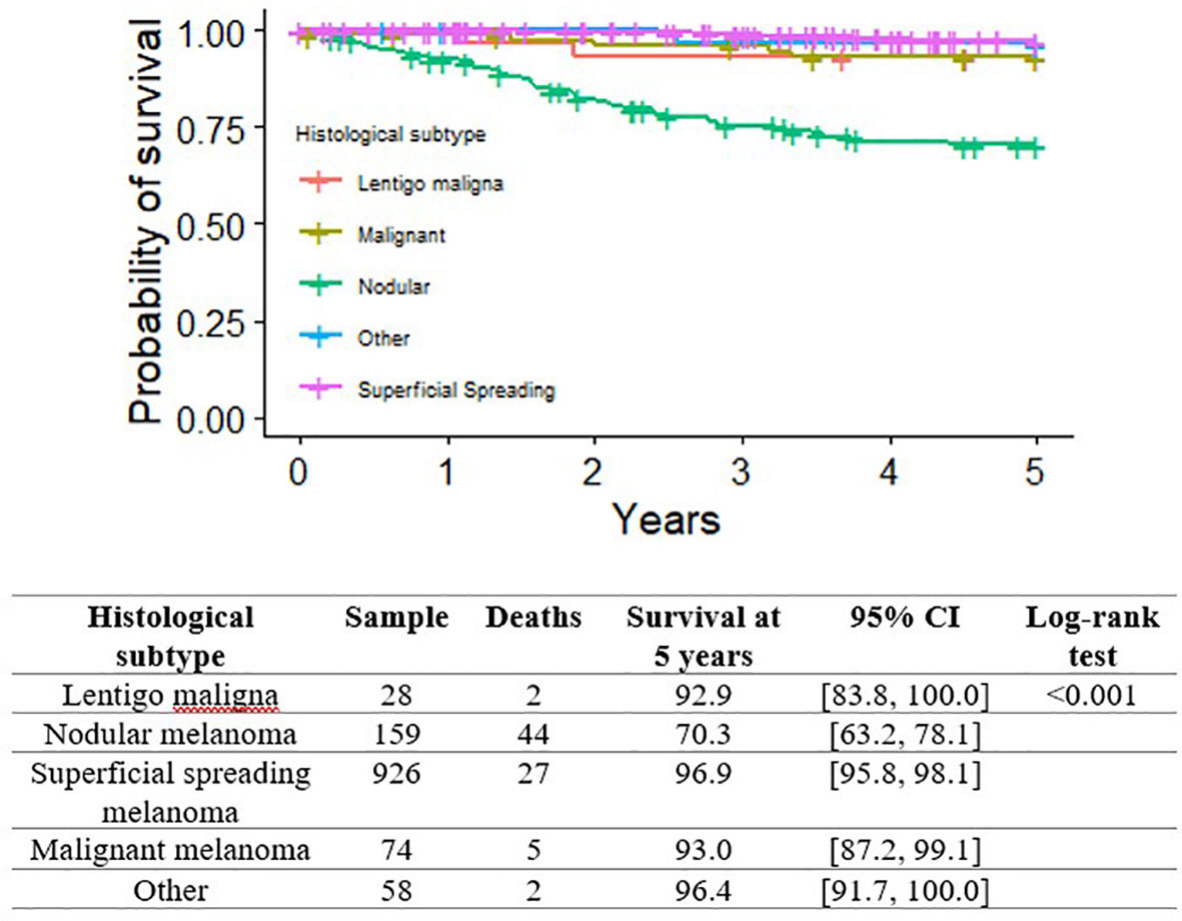
Kaplan-Meier curves for melanoma-specific survival by histological subtype and presence of mitoses.

**Figures 2**, **3** show the Kaplan-Meier MSS curves by each of the pathological variables considered at initial diagnosis (histological subtype, growth phase, mitotic index, ulceration, TILs). The 5-year MSS probability was 99.2% for the category 0-2 mitoses (95% CI: 98.6-99.8), and 76.2% (95% CI: 70.9-82.0) for more than 2 mitoses. Melanoma ulceration significantly affected the probability 5-year MSS (97.6%; 95%CI: 96.7-98.6 without ulceration *versus* 72.5%; 95% CI: 66.2-79.3). As for the tumor’s growth phase, survival was better for cases described as RGP (radial growth phase) at diagnosis than for those described as VGP (vertical growth phase): the 5-year MSS probability was 100.0% (95%CI: 100.0-100.0) for the former, and 91.6% (95%CI: 89.6- 93.8) for the latter. TIL status (presence *versus* absence) was associated with a small, but significant impact on 5-year MSS probability(94.4%, 95%CI: 92.9-95.9 *versus* 90.5%, 95%CI: 86.4-94.9, respectively). Finally, the survival analysis by histological subtype at diagnosis showed that nodular melanoma carried the worst 5-year MSS probability, at 70.3% (95%CI: 63.2-78.1). Superficial spreading melanoma had the highest 5-year MSS probability, at 96.9% (95% CI: 95.8-98.1). Intermediate survival probabilities were revealed for lentigo maligna melanoma (92.9%, 95% CI: 83.8-100.0).

**Table 2** shows the results of Cox’s regression model for MSS, adjusting for sex, age, histological subtype, ulceration, mitoses, site of tumor, stage at diagnosis and TILs. Compared with patients with a melanoma in stage I, the risk of death was increased for stage II (HR=3.31, 95% CI: 0.94-11.76, p=0.064), it was almost ten times higher for stage III (HR=10.51, 95% CI: 3.16-35.02, p<0.001), and it was more than a hundred times higher for stage IV (HR=117.17, 95% CI: 25.30-542.62, p<0.001). Superficial spreading melanoma carried a more than eleven times greater risk of death than lentigo maligna (HR=12.61, 95% CI: 1.42-112.02, p=0.023), and nodular melanoma a fourteen times higher risk (HR=15.04, 95% CI: 1.69-133.30, p=0.015). Sites of tumor involving the lower limbs, upper limbs and trunk had a better prognosis than those involving the hands and feet, with the difference reaching borderline statistical significance (p=0.058, p=0.083, p=0.066). Among the other variables included the model, the presence of mitoses emerged as an independent risk factor for death (HR=6.85, 95%CI: 2.21-21.28, p<0.001). The sensitivity analysis, excluding stage IV, generated much the same results as the previous model (data not shown). The analysis of overall survival produced similar results too, except that male sex coincided with a significantly worse prognosis (HR=1.75, % CI: 1.18-2.60, p=0.005).

**Table 2:** Cox’s regression analysis on cutaneous melanoma-specific survival patients, adjusting for sex, age, histological subtype, ulceration, mitotic count, CMM site, stage and TILs, as assessed at the patient’s enrolment.

		HR	95% CI	P value
**Sex**	Female	1.00	–	–
	Male	1.67	0.87 - 3.21	0.120
**Age**	<40	1.00	–	–
	40-49	1.45	0.17 - 13.55	0.743
	50-59	2.19	0.24 - 20.32	0.489
	60-69	3.28	0.41 - 26.39	0.265
	70-79	7.95	1.02 - 61.91	0.048
	80 or more	3.58	0.43 - 29.79	0.238
**CMM site**	Hands/feet	1.00	–	–
	Lower limbs	0.34	0.11 - 1.04	0.058
	Upper limbs	0.34	0.10 - 1.15	0.083
	Head	1.83	0.62 - 5.40	0.272
	Hands/feet	1.00	–	–
	Trunk	0.39	0.15 - 1.06	0.066
**CMM Histological subtype**	Lentigo maligna	1.00	–	–
	Nodular m.	15.04	1.69 - 133.30	0.015
	Superficial spreading m.	12.61	1.42 - 112.02	0.023
	NOS cutaneous m.	6.07	0.46 - 79.67	0.170
	Others	3.26	0.16 - 67.66	0.444
**CMM Ulceration**	Present	1.00	–	–
	Absent	0.82	0.41 - 1.62	0.562
**CMM Mitotic number**	0-2	1.00	–	–
	>2	6.85	2.21 - 21.28	<0.001
**CMM TILs**	Absent	1.00	–	–
	Present	1.70	0.80 - 3.59	0.166
**CMM TNM stage**	I	1.00	–	–
	II	3.31	0.94 - 11.76	0.064
	III	10.51	3.16 - 35.02	<0.001
	IV	117.17	25.30 - 542.62	<0.001

CMM, cutaneous melanoma; NOS, not otherwise specified; TILs, tumor infiltrating lymphocytes; m, melanoma. HR, hazard ratio; Assumption of proportionality: p-value 0.577.

## Discussion

In a population-based cohort of 1,279 incident CMM patients, this study focuses on the prognostic impact of both demographics and clinical-pathological variables, as recorded in a high-resolution Italian cancer registry.

The results obtained prompt two main types of consideration: one refers to the validation of the CMM-associated prognostic variables in a large cohort of consecutive patients; the other relates to the value of population-based trials for the purpose of updating/improving patient management based on a critical analysis of real-world clinical practice.

As regards the first point, the present results support the prognostic impact of (mostly) well-established clinical-pathological variables (6, 14, 15). In particular, the Kaplan-Meier analysis showed that none of the RGP CMMs resulted in a melanoma-specific death within 5 years after the initial diagnosis (16). The present results also provide evidence to show that extra-nodal metastases from RGP CMMs are extremely rare (less than 3%), while almost all extra-nodal metastatic implants result from “vertically-growing” CMMs (17). Consistently with these findings, both the worst MSS rate and the highest risk of CMM-related death were associated with nodular CMMs. Based on the assumption that any greater risk associated with a nodular histology overlaps with the prognostic impact of a melanoma’s thickness and ulceration, the American Joint Committee on Cancer (AJCC)’s staging system does not include the CMM subtype among the “discriminating” prognostic variables (14, 15). A recent analysis of the SEER cohort (18) nonetheless identifies the histological subtype as an independent predictor of survival, even after adjusting for CMM stage, thickness, ulceration, and mitotic index.

Previous studies found that the mitotic rate (more than neoplastic ulceration) is an independent prognostic factors in primary CMMs (irrespective of their thickness) (19–26). The present results associate a number of mitoses with a worse survival, further supporting the inclusion of the mitotic rate in the staging of thin, non-ulcerated CMMs.

A high-resolution cancer registry primarily needs to contain comprehensive, reliable, and accessible clinical information. All these conditions are hard to achieve, and the present study is no exception. In fact, our study suffered from the difficulty of assembling the necessary clinicopathological data, largely because of inconsistencies in the data format and/or their location in different digital repositories. The present study also suffers from a lack of important information on patients’ socio-economic profiles and - even more important - data on the molecular biology profile of the malignancies considered (27). In this respect, the present study further supports the crucial importance of promoting standardized/synoptic formats in the recording of clinicopathological variables, as obtained by the main clinical actors involved in patient management (especially oncologists, radiologists, and clinical and surgical pathologists).

Inconsistencies in the recording of diagnostic procedures and the “scattering” of results in different datasets represent major limits to operative efforts to pursue the high-resolution cancer registration potentially capable of providing both clinicians and healthcare policy-makers with reliable information on the clinical management of CMM patients.

## Data Availability Statement

The data analyzed in this study is subject to the following licenses/restrictions: The dataset generated as part of the present study is not publicly available, but available from the corresponding author (alessandra.buja@unipd.it) on reasonable request. Requests to access these datasets should be directed to alessandra.buja@unipd.it.

## Ethics Statement

The studies involving human participants were reviewed and approved by Veneto Oncological Institute Ethics Committee (n 695/20.10.2016). Written informed consent for participation was not required for this study in accordance with the national legislation and the institutional requirements.

## Author Contributions

ABu and GD conceived the presented idea. MZ, RS, AV, PF, and SM collected the data, verified its accuracy, and developed the methods. CT verified the analyses. ABa, ABu, and RF wrote the draft. MR, VB, GD, and ABu. supervised the project and revised the draft. CR found the financial support for the project. All authors read and approved the final manuscript.

## Funding

CARIPARO - Fondazione Cassa di Risparmio di Padova e Rovigo.

## Conflict of Interest

The authors declare that the research was conducted in the absence of any commercial or financial relationships that could be construed as a potential conflict of interest.

## Publisher’s Note

All claims expressed in this article are solely those of the authors and do not necessarily represent those of their affiliated organizations, or those of the publisher, the editors and the reviewers. Any product that may be evaluated in this article, or claim that may be made by its manufacturer, is not guaranteed or endorsed by the publisher.
